# Continuous Renal Replacement Therapy (CRRT) for Nonrenal Indications among Critically Ill Children with Malignancy

**DOI:** 10.1155/2021/6660466

**Published:** 2021-03-13

**Authors:** Wun Fung Hui, Kam Lun Hon, Alexander K. C. Leung, Karen Ka Yan Leung, Shu Wing Ku, Frankie W. T. Cheng

**Affiliations:** ^1^Department of Paediatrics and Adolescent Medicine, The Hong Kong Children's Hospital, Hong Kong; ^2^Department of Paediatrics, The University of Calgary and Alberta Children's Hospital, Calgary, Alberta T2M 0H5, Canada

## Abstract

The role of continuous renal replacement therapy (CRRT) has been expanding beyond support for acute kidney injury (AKI) in recent years. Children with malignancy are particularly at risk of developing conditions that may require CRRT. We reported three children with malignancy who received CRRT for non-AKI indications. Patient 1 was a 17-year-old teenage girl who developed refractory type B lactic acidosis due to relapse of acute lymphoblastic leukemia (ALL). Her peak lactate level was 18 mmol/L, and the lowest pH and bicarbonate level was 7.13 and 6.0 mmol/L, respectively. She received three sessions of high-volume hemodiafiltration to bring down the lactate level. Patient 2 was a 15-year-old male with T-cell ALL who developed cytokine storm requiring mechanical ventilatory and high-dose inotropic support due to necrotizing enterocolitis complicated by pneumoperitoneum and *Klebsiella pneumoniae* septicemia. He received two sessions of hemoperfusion using a specific filter capable of endotoxin absorption and cytokine removal and was successfully weaned off all inotropes after the treatment. Patient 3 was an 8-year-old boy who received bone marrow transplantation and developed worsening hyperbilirubinemia and deteriorating liver function. He received a session of single-pass albumin dialysis for bilirubin removal prior to liver biopsy. Except for mild electrolyte disturbances, no major CRRT complication was encountered. Our report demonstrated that CRRT is an effective and safe procedure for a wide spectrum of nonrenal conditions among children with oncological diagnoses in the pediatric intensive care unit. However, the optimal dose, regime, timing of initiation, and monitoring target for these indications remain to be determined.

## 1. Introduction

Since the first continuous renal replacement therapy (CRRT) that was performed in 1977, it has become the most popular mode of acute renal replacement therapy for critically ill adults and children [1, 2]. With the advancement of technology and availability of tailor-made equipment dedicated for pediatric patients, CRRT can now be carried out smoothly and safely in children [1]. While acute kidney injury (AKI) and its associated complications have remained one of the most important indications for CRRT, CRRT has also been used for treatment of various non-AKI conditions in recent years. The US Prospective Pediatric Continuous Renal Replacement Therapy (ppCRRT) registry reported that, from 2001 to 2005, CRRT was performed on 50 subjects for nonrenal indications (14.5% of the whole registry) including inborn error of metabolism (IEM) (42%), drug intoxication (36%), and tumor lysis syndrome (TLS) (22%) [3]. Besides, different techniques of extracorporeal blood purification and the addition of sorbent for removal of toxic substances have widened the application of CRRT.

Children with malignancy are at risk of developing various conditions such as TLS, toxic effect of chemotherapy, and AKI with sepsis that may require CRRT at presentation or during their course of treatment [4, 5]. We hereby describe our experience of employing CRRT and extracorporeal treatment for different conditions in children with malignancy in the pediatric intensive care unit (PICU) of a newly established children's hospital.

## 2. Case Series

### 2.1. Patient 1

**Figure 1:**
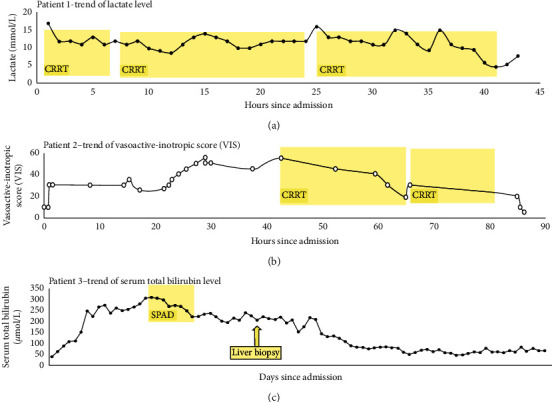
The effectiveness of continuous renal replacement therapy (CRRT) of the three patients.

A 17-year-old girl with a history of B-cell acute lymphoblastic leukemia (ALL) was admitted for generalized malaise, poor appetite, and weight loss over 6 weeks. Her initial blood investigations revealed hemoglobin 11.3 g/dL, white blood cell 3.7 × 10^9^/L, and platelet 83 × 10^9^/L with no blast identified. Her urea and creatinine levels were normal, but she had mild electrolyte disturbances with a serum potassium level of 3.3 mmol/L, phosphate level of 0.68 mmol/L, and uric acid level of 0.42 mmol/L. She also had severe lactic acidosis with a blood pH 7.18, pCO2 3.8, bicarbonate 10.3 mmol/L, base excess −10.0 mmol/L, and lactate 15 mmol/L. She was given bicarbonate infusion at a concentration of 30 mmol/L and was hemodynamically stable upon PICU admission. Bone marrow examination confirmed relapse of ALL, and no metabolic or toxicological cause was identified for the lactic acidosis. The bicarbonate infusion was stepped up to 100 mmol/L; however, the lactic acidosis persisted with a peak lactate level 18 mmol/L, lowest pH 7.13, and bicarbonate level 6.0 mmol/L. Owing to the refractory metabolic acidosis, she was started on high-volume hemodiafiltration for lactate removal (Table 1). She received altogether 160 hours of CRRT with a peak dialysate flow rate of 89.8 ml/kg/hour. The maximal prescribed dose (replacement flow plus dialysate flow) was 111 ml/kg/hour. The mean lactate clearance achieved was 65 ml/kg/hour. She experienced transient hypotension at the initiation of CRRT which spontaneously recovered. There was mild hypokalemia, hypophosphatemia, and hypomagnesemia during the treatment. Otherwise, no major complication was encountered. Her lactate level slowly decreased (Figure 1), and the lactate level after CRRT treatment was 7.8 mmol/L. She was discharged from PICU 11 days after admission.

### 2.2. Patient 2

A 15-year-old teenage male with T-cell ALL receiving active chemotherapy treatment developed intermittent vomiting associated with fever and abdominal pain. Subsequent investigations confirmed the diagnosis of peritonitis with pneumoperitoneum. His white blood cell and neutrophil count was 3.23 × 10^9^/L and 2.54 × 10^9^/L, respectively. His serum lactate level was 4.8 mmol/L, C-reactive protein (CRP) level was 134 mg/L, and procalcitonin level was 45.1 ng/ml. He was immediately given fluid resuscitation and started on broad-spectrum empirical antimicrobials, namely, vancomycin, meropenem, metronidazole, and micafungin following sepsis workup. An urgent laparotomy revealed multiple segments of dusky and necrotic bowel requiring resection of 40 cm of small bowel. Part of the bowel with doubtful viability was also resected. He developed hypotension requiring adrenaline infusion and phenylephrine during the operation. Postoperatively, he was maintained on mechanical ventilatory and inotropic support. Adrenaline was then changed to noradrenaline, and amikacin was added for antimicrobial synergistic effect. The blood culture and peritoneal swab both isolated *Klebsiella pneumoniae* that was sensitive to meropenem and gentamicin. The pathology of the resected bowel showed features of necrotizing enterocolitis with mixed Gram-positive and Gram-negative cocci and bacilli in both the bowel lumen, muscle layer, and the serosa. One dose of intravenous immunoglobulin (1 gm/kg) was given one day after the operation. However, the condition of the patient remained critical. There was persistent fever, and his lactate level remained at 4–6 mmol/L. He also developed thrombocytopenia and disseminated intravascular coagulopathy, and the dosages of inotropes were escalating. Hence, hemoperfusion using the Oxiris^®^ filter was started for cytokine removal and endotoxin absorption (Table 1). His urine output 24 hours prior to initiation of CRRT was 3.7 ml/kg/hour, and the degree of fluid overload as determined by (fluid in–fluid out (L))/PICU admission body weight (kg) ×100% was +1.4%. The vasoactive-inotropic score (VIS) at CRRT initiation was 55. He received two sessions of hemoperfusion lasting 24 hours and 13 hours, respectively. Except for mild hypophosphatemia and hypomagnesemia, there was no CRRT-related complication. The inotropic doses were reduced rapidly afterwards (Figure 1), and lactate, CRP, and procalcitonin levels also decreased. He was stabilized, and a second operation was performed with the remaining necrotic bowel resected and the small bowel exteriorized. He was successfully extubated, and all the inotropes were weaned off 3 days after the initiation of the CRRT.

### 2.3. Patient 3

An 8-year-old boy received bone marrow transplantation due to relapse of B-cell ALL. The patient developed veno-occlusive disease (VOD) and graft-versus-host disease (GVHD) of the lung and gut and was treated with mycophenolate mofetil and methylprednisolone. He had an episode of fluid retention due to the VOD resulting in acute pulmonary edema requiring mechanical ventilation and CRRT for fluid removal. Thereafter, he evolved to develop hepatorenal syndrome with hyperbilirubinemia and remained CRRT-dependent. The serum total bilirubin level slowly increased from 40 *μ*mol/L to >250 *μ*mol/L over 2 weeks despite various medications and liver-protective total parenteral nutrition. There was associated hyperammonemia and elevated liver enzymes. Sixteen days after PICU admission, the total and conjugated bilirubin level reached a peak level of 305 *μ*mol/L and 263 *μ*mol/L, respectively. After discussion with an oncologist, surgeons, and an interventional radiologist, a liver biopsy was planned to delineate the exact pathology of the deteriorating liver function. A session of single-pass albumin dialysis (SPAD) using 4% albumin as dialysate was performed for bilirubin removal before the liver biopsy (Table 1). The patient received 42 hours of treatment altogether with the mean dialysate flow rate achieved at 30.3 ml/kg/hour. Except for asymptomatic mild electrolytes disturbances, there was no major SPAD-related complication. The bilirubin dropped gradually and the total and direct bilirubin level after the therapy was 222 and 191 *μ*mol/L, respectively (Figure 1). The therapy bridged him for liver biopsy, which confirmed to be hepatic acute VOD together with GVHD. Tacrolimus was then added, and the bilirubin gradually subsided.

## 3. Discussion

Our three cases have demonstrated that CRRT can be utilized effectively and safely in a wide spectrum of conditions among children with malignancy. The characteristics of children may be different between those receiving CRRT for renal and nonrenal indications, and the regime of CRRT may vary according to the specific indication and the characteristic of the substances to be removed.

The definitive treatment of malignancy-associated type B lactic acidosis should be specific therapy targeting the underlying malignancy. Type B lactic acidosis is not associated with tissue hypoxia or hypoperfusion. Extracorporeal lactate removal may act as an interim therapy before chemotherapy becomes effective. Lactate is a small molecule with a molecular weight of 90 daltons, making it favorable for dialysis clearance. In fact, a number of case reports have demonstrated the effectiveness of CRRT for lactate removal in various conditions [6–8]. Although the clearance of CRRT may not be comparable to hemodialysis, the ongoing production of lactate may favor the use of CRRT. A high dose of dialysate component is required for clearance of toxic metabolite with small molecular weights using CRRT, especially for neurotoxic substances such as ammonia as the duration of exposure to high level of toxic metabolite is crucial to the long-term outcome [9, 10]. We also employed a very high dialysate flow in our patient. Although we were able to bring down the lactate level with such high-volume CRRT, the lactate level attained was still higher than normal, and this could be accounted by the ongoing lactate production exceeding the clearance. The optimal CRRT dose and criteria to initiate and discontinue CRRT for lactic clearance is still unclear.

The role of extracorporeal blood purification among patients with sepsis and AKI has gained much interest in recent years. The systemic inflammatory response syndrome triggered by the endotoxin and proinflammatory cytokines in sepsis contributes to early mortality [11]. Hence, early extracorporeal removal of endotoxin and cytokines can be a potential strategy for sepsis management. As endotoxin is a large molecule that cannot pass through the hemofilter, adsorption by means of hemoperfusion is required for its removal. The use of a filter capable of endotoxin adsorption and cytokines removal has attracted much attention [12, 13], especially with the COVID-19 pandemic [14]. The specific filter used in our patient is a 3-layered AN69-based membrane. An *in vitro* experiment showed that it can effectively reduce endotoxin level and improve hemodynamic parameters in the septic porcine model [15]. The number of reported cases in pediatric patients is scarce and is mostly limited to adolescent [16]. This is probably related to the lack of suitable equipment tailor made for small children. The surface area of this filter is 1.5 m^2^ and it is recommended to be used in patients weighting >30 kg. A recently reported crossover clinical trial that involved 16 adult patients with sepsis, AKI, and elevated endotoxin level using either Oxiris® or standard filter showed a significant reduction in the endotoxin, lactate, and cytokine levels and noradrenaline dose in the Oxiris® group [17]. The baseline lactate, endotoxin, and cytokine levels were higher in the Oxiris® group and patient outcome was not reported [17]. CytoSorb® and Toraymyxin^TM^ are commercially available alternatives for cytokine removal and endotoxin adsorption, respectively. CytoSorb® is an adsorption column made of biocompatible porous polymer beads, and Toraymyxin^TM^ is a polymyxin B-immobilized fibers blood purification column. Detailed review and comparison of different sorbent therapies is beyond the scope of this report. Although extracorporeal endotoxin and cytokines removal may serve as an adjunctive therapy in sepsis management, currently the optimal timing of initiation, the monitoring target, and dose of therapy are largely unknown. We were not able to measure endotoxin or cytokines level for our patient and used clinical parameters to guide the duration of therapy for our patient. One of the targets would be to reduce the dose of inotropes provided hemodynamic parameters permit. As the number of studies relating to clinical outcome especially in children is scarce, prospective study is needed to better define the role of CRRT filter in the management of sepsis.

The technique of single-pass albumin dialysis (SPAD), which is very similar to CRRT, involves the use of standard CRRT machines and circuit using albumin as the dialysate solution to remove the albumin-bound toxins such as bilirubin and bile acid or medication with high degree of protein binding. It is therefore employed as a bridging therapy for liver failure [18] and as a treatment modality for drug intoxication [19]. *In vitro* study has demonstrated the effective clearance of bilirubin using SPAD and higher efficacy can be achieved with a higher flow rate and a more concentrated albumin solution [20]. In fact, it is useful to remove substances with a molecular weight up to 60,000 daltons [21]. A prospective crossover trial has also demonstrated comparable efficacy in bilirubin removal comparing SPAD and the commercially available MARS (molecular adsorbents recirculating system) [22]. However, the clearance of bile acid was inferior for SPAD compared to MARS. The data on clinical application of SPAD in children are scarce, and the timing of initiation and optimal dose of using SPAD in various conditions are not well studied. However, it appeared to be a relatively uncomplicated and safe procedure in the intensive care setting with favorable patient outcomes [18, 23]. Nevertheless, the therapy itself is labor intensive if online albumin solution generation is not available, and the cost of using a large amount of albumin is considerable.

## 4. Conclusion

CRRT has evolved to be an indispensable treatment for a wide spectrum of conditions,. We have demonstrated the safe and effective use of CRRT for non-AKI indications in children with malignancy. The optimal dosing, CRRT regime, and monitoring targets for these conditions are still unknown and will probably vary between different indications. However, a high-volume hemodiafiltration for metabolite removal and early initiation of hemoperfusion and sorbent therapy for sepsis appear to be a rational approach. Further prospective study is in great need to better define the timing, regimes, and techniques of CRRT  for different indications in children.

## Figures and Tables

**Table 1:** Clinical characteristics and continuous renal replacement therapy (CRRT) parameters of the three patients.

Variable	Patient 1	Patient 2	Patient 3
Clinical characteristics			
Age (year)	17.1	15.4	8.5
Sex	Female	Male	Male
Indication for CRRT	Lactic acidosis	Sepsis/cytokine storm	Hyperbilirubinaemia
Pre-CRRT fluid overload (%)	−1.6% (over 12 hours)	+1.4% (over 24 hours)	−2.6% (over 24 hours)
Pre-CRRT urine output (ml/kg/hour)	3.8 (over 12 hours)	3.7 (over 24 hours)	0.04 (over 24 hours)
PIM3 predicted mortality (%)	6.2	24.2	5.9
Pre-CRRT creatinine (*μ*mol/L)	29	45	55
Pre-CRRT eGFR (ml/min/1.73 m^2^)	210.2	133.8	45.6
Need for mechanical ventilation	No	Yes	No
Need for inotropes	No	Yes	No

CRRT parameters			
Technique	High-volume hemodiafiltration	Hemoperfusion	Single-pass albumin dialysis
Time to therapy (hour)	11.9	48	203.8
Filter	ST100	Oxiris®	ST100
Mean blood flow rate (ml/kg/min)	4.7	4.1	3.7
Mean replacement rate (ml/kg/hour)	21.4	32.5	23.0
Mean dialysate rate (ml/kg/hour)	72.7	32.2	30.3
Mean dose^*∗*^ (ml/kg/hour)	94.8	56.6	59.2
Maximal dose (ml/kg/hour)	111.1	73.2	60.0
Anticoagulation	Heparin	Nil	Nil
Treatment duration (hour)	160.9	37.0	42.1

^*∗*^Dose = replacement rate + dialysate rate; eGFR: estimated glomerular filtration rate; PIM3: Pediatric Index of Mortality 3.

## References

[1] Ronco C. (2017). Continuous renal replacement therapy: forty-year anniversary. *The International Journal of Artificial Organs*.

[2] Digvijay K., Neri M., Fan W., Ricci Z., Ronco C. (2019). International survey on the management of acute kidney injury and continuous renal replacement therapies: year 2018. *Blood Purification*.

[3] Fleming G. M., Walters S., Goldstein S. L. (2012). Nonrenal indications for continuous renal replacement therapy. *Pediatric Critical Care Medicine*.

[4] Park P. G., Hong C. R., Kang E. (2019). Acute kidney injury in pediatric cancer patients. *The Journal of Pediatrics*.

[5] Fiser R. T., West N. K., Bush A. J., Sillos E. M., Schmidt J. E., Tamburro R. F. (2005). Outcome of severe sepsis in pediatric oncology patients. *Pediatric Critical Care Medicine*.

[6] Hui W. F., Luk C. W., Chan W. K. Y., Miu T. Y., Yuen H. L. (2012). Severe lactic acidosis in an infant with haemophagocytic lymphohistiocytosis. *HongKong Journal of Paediatr (New Series)*.

[7] Cheungpasitporn W., Zand L., Dillon J. J., Qian Q., Leung N. (2015). Lactate clearance and metabolic aspects of continuous high-volume hemofiltration. *Clinical Kidney Journal*.

[8] Keller G., Cour M., Hernu R., Illinger J., Robert D., Argaud L. (2011). Management of metformin-associated lactic acidosis by continuous renal replacement therapy. *PLoS One*.

[9] Jouvet P., Schaefer F., Warady B. A., Alexander S. R., Schaefer F. (2012). Dialytic therapy for inborn errors of metabolism. *Pediatric Dialysis*.

[10] Hanudel M., Avasare S., Tsai E., Yadin O., Zaritsky J. (2014). A biphasic dialytic strategy for the treatment of neonatal hyperammonemia. *Pediatric Nephrology*.

[11] Moreland J. G., Wheeler D., Wong H., Shanley T. (2014). The immune system in critical illness and injury. *Pediatric Critical Care Medicine*.

[12] Bottari G., Di Nardo M., Gleeson J. (2019). Extracorporeal blood purification techniques in children with hyper-inflammatory syndromes: a clinical overview. *Minerva Anestesiologica*.

[13] Girardot T., Schneider A., Rimmelé T. (2019). Blood purification techniques for sepsis and septic AKI. *Seminars in Nephrology*.

[14] Ronco C., Bagshaw S. M., Bellomo R. (2020). Extracorporeal blood purification and organ support in the critically ill patient during COVID-19 pandemic: expert review and recommendation. *Blood Purification*.

[15] Rimmele´ T., Assadi A., Cattenoz M. (2009). High-volume hemofiltration with a new hemofiltration membrane having enhanced adsorption properties in septic pigs. *Nephrol Dial Transplant*.

[16] Hui W. F., Chan W. K. Y. (2017). Role of hemofilter with endotoxin adsorption capacity in management of septic shock. *Critical Care Shock*.

[17] Broman M. E., Hansson F., Vincent J. L., Bodelsson M. (2019). Endotoxin and cytokine reducing properties of the oXiris membrane in patients with septic shock: a randomized crossover double-blind study. *PLoS One*.

[18] Ringe H., Varnholt V., Zimmering M. (2011). Continuous veno-venous single-pass albumin hemodiafiltration in children with acute liver failure. *Pediatric Critical Care Medicine*.

[19] Chan W. K. Y., Hui W. F. (2016). Sequential use of hemoperfusion and single-pass albumin dialysis can safely reverse methotrexate nephrotoxicity. *Pediatric Nephrology*.

[20] Peszynski P., Klammt S., Peters E. (2002). Albumin dialysis: single pass vs. recirculation (MARS). *Liver*.

[21] Ghannoum M., Roberts D. M., Hoffman R. S. (2014). A stepwise approach for the management of poisoning with extracorporeal treatments. *Seminars in Dialysis*.

[22] Sponholz C., Matthes K., Rupp D. (2016 4). Molecular adsorbent recirculating system and single-pass albumin dialysis in liver failure--a prospective, randomised crossover study. *Crit Care*.

[23] Holle J., Gratopp A., Balmer S. (2020). Single-pass albumin dialysis in the treatment of children with liver failure. *Blood Purif*.

